# High levels of X-linked Inhibitor-of-Apoptosis Protein (XIAP) are indicative of radio chemotherapy resistance in rectal cancer

**DOI:** 10.1186/s13014-015-0437-1

**Published:** 2015-06-13

**Authors:** L. Flanagan, J. Kehoe, J. Fay, O. Bacon, A.U. Lindner, E.W. Kay, J. Deasy, D.A. McNamara, J.H.M. Prehn

**Affiliations:** Department of Physiology and Medical Physics, Royal College of Surgeons in Ireland, 123 St. Stephen’s Green, Dublin 2, Ireland; Centre for Systems Medicine, Royal College of Surgeons in Ireland, 123 St. Stephen’s Green, Dublin 2, Ireland; Departments of Pathology, Beaumont Hospital, Dublin 9, Ireland; Departments of Surgery, Beaumont Hospital, Dublin 9, Ireland

**Keywords:** Rectal cancer, Neoadjuvant radio chemotherapy, Apoptosis, Inhibitor of apoptosis proteins

## Abstract

**Background:**

The mainstay of treatment in rectal cancer is neoadjuvant radio chemotherapy prior to surgery, in an attempt to downstage the tumour, allowing for more complete removal during surgery. In 40 % of cases however, this neoadjuvant radio chemotherapy fails to achieve tumour regression, partly due insufficient apoptosis signaling. X-linked Inhibitor of Apoptosis Protein (XIAP) is an anti-apoptotic protein that has been reported to contribute to disease progression and chemotherapy resistance.

**Methods:**

We obtained rectal biopsy normal and matched tumour tissue from 29 rectal cancer patients with varying degrees of tumour regression, and using Western blot, examined anti-apoptotic XIAP and pro-apoptotic Smac protein levels in these tissues, with the aim to examine whether disturbed XIAP/Smac levels may be an indicator of neoadjuvant radio chemotherapy resistance. Expression of inhibitor of apoptosis proteins cIAP-1 and cIAP-2 was also examined.

**Results:**

We found that levels of XIAP increased in accordance with the degree of radio chemotherapy resistance of the tissue. Levels of this protein were also significantly higher in tumour tissue, compared to matched normal tissue in highly resistant tissue. In contrast, Smac protein levels did not increase with radio chemotherapy resistance, and the protein was similarly expressed in normal and tumour tissue, indicating a shift in the balance of these proteins. Post treatment surgical resection tissue was available for 8 patients. When we compared matched tissue pre- and post- radio chemotherapy we found that XIAP levels increased significantly during treatment in both normal and tumour tissue, while Smac levels did not change. cIAP-1 and cIAP-2 levels were not differentially expressed in varying degrees of radio chemotherapy resistance, and neoadjuvant therapy did not alter expression of these proteins.

**Conclusion:**

These data indicate that disturbance of the XIAP/Smac balance may be a driver of radio chemotherapy resistance, and hence high levels of XIAP may be a useful indicator of neoadjuvant radio chemotherapy resistance in rectal cancer. Moreover, as XIAP levels increase with radio chemotherapy it is possible that a subset of more resistant tumour cells survive this treatment and may be resistant to further adjuvant treatment. Patients with resistant tumours highly expressing XIAP may benefit from alternative treatment strategies, such as Smac mimetics post neoadjuvant radio chemotherapy.

## Background

Under homeostatic conditions a balance is struck between cell survival and cell death. Dysregulation of proteins involved in apoptosis, or programmed cell death, can upset this balance and push cells towards a disease state. Excessive apoptosis can lead to neurodegenerative diseases, and conversely situations where apoptosis is deficient can result in carcinogenesis and chemo resistance [1–4]. In the rectal cancer treatment setting neoadjuvant radio chemotherapy is performed prior to surgery in the hope of downsizing the tumour, allowing for more complete removal during surgery [5]. Responses to neoadjuvant radio chemotherapy can vary greatly between patients, and are categorised according to the Royal College of Pathologists depending on extent of tumour regression as RCPath A (complete tumour regression), RCPath B (partial tumour regression), or RCPath C (no marked tumour regression) [6]. In 15–27 % of patients therapy is successful and a complete pathological response is achieved, with patients displaying no residual tumour [7–9]. On the other hand, in 30–40 % of patients no tumour regression is seen following neoadjuvant radio chemotherapy [9, 10]. Molecular biomarkers indicative of treatment response could help streamline treatment selection and spare some patients from undergoing inefficient treatments.

Defective or deregulated apoptosis may be the driving force behind this chemo resistance. X-linked Inhibitor of Apoptosis Protein (XIAP) is an anti-apoptotic protein that acts to suppress cell death via potent inhibition of caspases [11–13]. High expression of this protein corresponds to poor prognosis in many cancers [14–16], and furthermore its expression level correlates positively with disease progression [17]. As well as contributing to disease progression, XIAP was also reported to contribute to chemotherapy resistance, and targeting this protein was found to effectively sensitise cells to apoptosis and suppress tumour progression [18–21]. Second mitochondria-derived activator of caspases (Smac) is a potent inhibitor of XIAP. By binding to XIAP, Smac relieves caspase inhibition and allows apoptosis to proceed. Cytosolic Smac also binds to cIAP-1 and-2, inducing rapid autoubiquitination and proteasomal degradation of Smac and cIAPs, resulting in an NF-κB-dependent secretion of TNF-α and subsequent autocrine apoptosis induction [22–24]. In cancer cells where XIAP is over expressed Smac levels may be insufficient to block XIAP activity, and this disturbed XIAP/Smac balance may contribute to apoptotic resistance. Studies demonstrate that restoration of the XIAP/Smac balance in chemotherapy resistant cancer cells can re-establish apoptosis [25, 26]. As a result synthetic Smac peptides are being investigated for their therapeutic value in cancer. In conjunction with other drugs, such as TRAIL, these compounds have been successful in sensitising cancer cells to apoptosis and are currently in clinical trials [27].

A better understanding of the molecular changes occurring during neoadjuvant radio chemotherapy could help identify markers of response, or tools to monitor treatment response. Here we performed a quantitative analysis of XIAP and Smac protein levels in the pretreatment biopsy tissue of a cohort of rectal cancer patients displaying variable responses to neoadjuvant radio chemotherapy, with the aim of studying whether disturbed XIAP/Smac levels are indicative of resistance to neoadjuvant radio chemotherapy. Furthermore we examined matched pre and post treatment tissue to detect changes in XIAP or Smac expression induced by therapy. We also examined cIAP-1 and cIAP-2 expression in this tissue to examine whether these proteins may play a contributory role when XIAP/Smac balance is disturbed.

## Materials and methods

### Patient cohort

Both biopsy and resection patient tissue was obtained from the Departments of Surgery, and Pathology, Beaumont Hospital, Dublin, Ireland. At colonoscopy or rigid sigmoidoscopy biopsy tumour and matched normal samples were collected. Biopsy tissue was collected in 21 male and 8 female patients (Table 1). The median age of patients was 65, with ages ranging from 38 to 79. To ensure consistent quality and tumour presence both normal and tumour tissues were evaluated by an experienced pathologist. Normal samples were obtained from a distant site in the rectal tract that was macroscopically unaffected and disease free. To be included in our analysis tumour samples must have contained over 50 % tumour cells and normal samples must have been free of malignant cells. Following colonoscopy or rigid sigmoidoscopy patients underwent radio chemotherapy. The radio chemotherapy regimens consisted of radiotherapy (50.4 GY in 28 fractions) and 5FU (5-Fluorouracil), but some regimes varied slightly depending on tolerance of the patient. Once patients completed their course of treatment they underwent surgical resection. Post-treatment surgical resection tissue was obtained during surgery. Resection tissues were examined by a pathologist and graded based on regression of the tumour in response to therapy. Complete tumour regression (RCPath A) was achieved in 5 patients; partial tumour regression (RCPath B) in 10 patients, and no marked tumour regression (RCPath C) was observed in 14 patients. A dedicated clinical research nurse reviewed medical records and gathered clinical information. Patients with a family history of colorectal cancer were excluded from analysis. Informed consent was obtained from all patients and Beaumont Hospital Ethics Committee granted ethical approval for the work.

**Table 1:** Table of patient clinical characteristics

ID	Sex	Age	Neoadjuvant therapy	RCPath	Resection tissue	Staging		
						T	N	M
1	M	73	50.4/28 + 5fu	A	x	3	2	0
2	F	42	54/30 + 5fu	A	x	2,3	1,2	0
3	F	38	50.4/28 + 5fu	A	x	3	1	x
4	M	75	54/31 + 5fu	A	x	3	2	x
5	M	51	50.4/28 + 5fu	A	x	3	2	0
6	M	66	50.4/28 + 5fu	B	√	3	0,1	0
7	M	57	50.4/28 + 5fu	B	√	3	1,2	0
8	F	72	50.4/28 + 5fu	B	x	3	2	0
9	M	65	50.4/28 + 5fu	B	x	3	x	x
10	M	65	50.4/28 + 5fu	B	x	3	2	x
11	F	64	42.2/20 + 5fu	B	x	2	0	0
12	F	60	50.4/28 + 5fu	B	x	2	0	x
13	M	66	50.4/28 + 5fu	B	x	3	2	0
14	M	67	50.4/28 + 5fu	B	x	3	1	0
15	M	67	54/30 + 5fu	B	x	3	1	x
16	F	55	55.4/30 + 5fu	C	√	3	2	0
17	F	75	45/25 + 5fu/RP	C	x	3	0	0
18	M	70	50.4/28 + 5fu	C	√	3	2	0
19	M	79	50.4/28 + 5fu	C	x	3	2	0
20	M	76	43.2/25 + 5fu	C	√	3	2	0
21	M	48	50.4/28 + 5fu	C	x	3	0	0
22	M	44	50.4/30 + 5fu	C	√	3	2	0
23	M	47	50.4/28 + 5fu	C	√	3	2	x
24	M	74	50.4/28 + 5fu	C	√	3	2	x
25	M	45	50.4/28 + 5fu	C	x	3	2	x
26	M	62	50.4/30 + 5fu	C	x	3	1	x
27	M	64	50.4/28 + 5fu	C	x	3	1	x
28	F	41	50.4/28 + 5fu	C	x	3	1	x
29	M	71	50.4/28 + 5fu	C	x	3	2	x

### Protein extraction and quantification

All tissue samples were lysed in 400 mL ice-cold buffer containing 50 mmol/L HEPES (pH 7.5), 150 mmol/L NaCl, 5 mmol/L Na-EDTA and protease inhibitor (Sigma). Samples were maintained on ice and homogenised using the Ultra-Turrax T25 Basic Homogeniser using 30 s pulses to break down the tissue. Protein concentrations were determined as follows, using the standard Pierce Micro-BCA Protein Assay (Pierce, Northumberland, UK): For calibration, a standard curve ranging from 0–12 μg was set up using a bovine serum albumin standard. Samples and standards were incubated at 37 °C for 30 min and then absorbance was measured at 560 nm. The average absorbances were calculated for each sample and standard. The slope of the BSA standard curve was used to calculate the protein concentration of each sample. Protein samples were prepared with SDS loading buffer (100 nM TRIS-Cl pH 6.8, 4 % SDS, 0.2 % bromophenol blue, 20 % glycerol) and denatured at 95 °C for 10 min. An equal amount of 20 μg protein was loaded onto each lane of 10–15 % SDS-polyacrylamide gels. Gels were run at a voltage of 80 V until the proteins had migrated through the stacking gel, and then at 120 V to drive the proteins through the resolving gel. The running buffer was composed of 25 mM Tris-Cl, pH 8.3, 250 mM glycine and 0.1 % SDS. Once the proteins had migrated through the gel the current was stopped and gels were transferred to Nitrocellulose membranes. Gels were blotted to nitrocellulose membranes (Protean BA 83; 2 μm; Schleicher & Schuell) in transfer buffer (25 mM Tris, 192 mM glycine, 20 % methanol (v/v), and 0.01 % SDS). Gels were transferred at 18 V for 60 min. The nitrocellulose membranes were then blocked with 5 % nonfat dry milk in TBST (15 mM Tris–HCl, pH 7.5, 200 mM NaCl, and 0.1 % Tween 20) at room temperature for 1 h. Membranes were then incubated with the primary antibodies overnight at 4 °C. The following primary antibodies were used: XIAP (1:1000; Mouse monoclonal, 610762, BD transduction laboratories, USA), Smac (1:1000; Rabbit polyclonal, AF-789 R&D Systems, USA), cIAP1 (1:1000; Rabbit polyclonal, #4952, CST), cIAP2 (1:1000; Rabbit monoclonal, #3130, CST) and b-actin (1:5000; Sigma). Secondary antibodies were horseradish peroxidase (HRP)-conjugated and were used at a dilution of 1:10,000 (Millipore), and membranes were incubated for 1 h at room temperature. Blots were developed using the enhanced chemiluminescence detection reagent (Millipore, Ireland). Chemiluminescence was detected at 12-bit dynamic range using a Fuji LAS 4000 CCD system (Fujifilm UK Ltd., Bedfordshire, UK). Densitometry was then performed. The intensity of each band was calculated using Image J software. The intensity of the loading control was deducted from the intensity of the band of interest to eliminate any differences in protein levels owing to uneven loading, so that each sample was normalized and could be compared. Each sample was normalised to the intensity of its corresponding β-Actin band.

### Statistics

Statistical analyses were performed in Matlab (MathWorks, Natick, MA, USA), and SPSS (IBM, Armonk, NY, USA). Data are given as means ± s.e.m. For statistical comparison Student’s *t* test or ANOVA and subsequent Tukey test were used for normal distributed data. *P* values ≤ 0.05 were considered statistically significant.

## Results

### XIAP level increases in accordance with tissue resistance to radio chemotherapy in tumour tissue, but not normal tissue

**Fig. 1:**
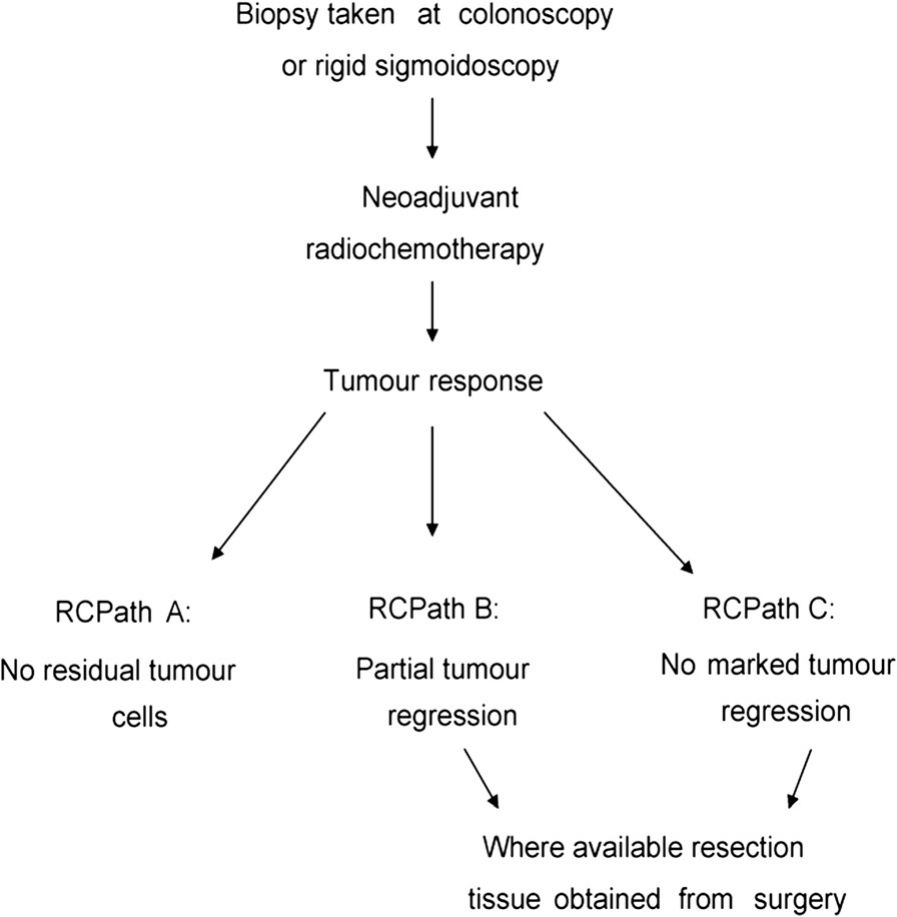
Sample collection and clinical response. Biopsy samples were obtained at colonoscopy or rigid sigmoidoscopy. Patients then underwent neoadjuvant radio chemotherapy. Patient responses are categorised depending on the degree of tumour regression following neoadjuvant radio chemotherapy as RCPath **A** (complete tumour regression), RCPath **B** (partial tumour regression), or RCPath **C** (no marked tumour regression). Following neoadjuvant radio chemotherapy, resection tissue was obtained from surgery where available

**Fig. 2:**
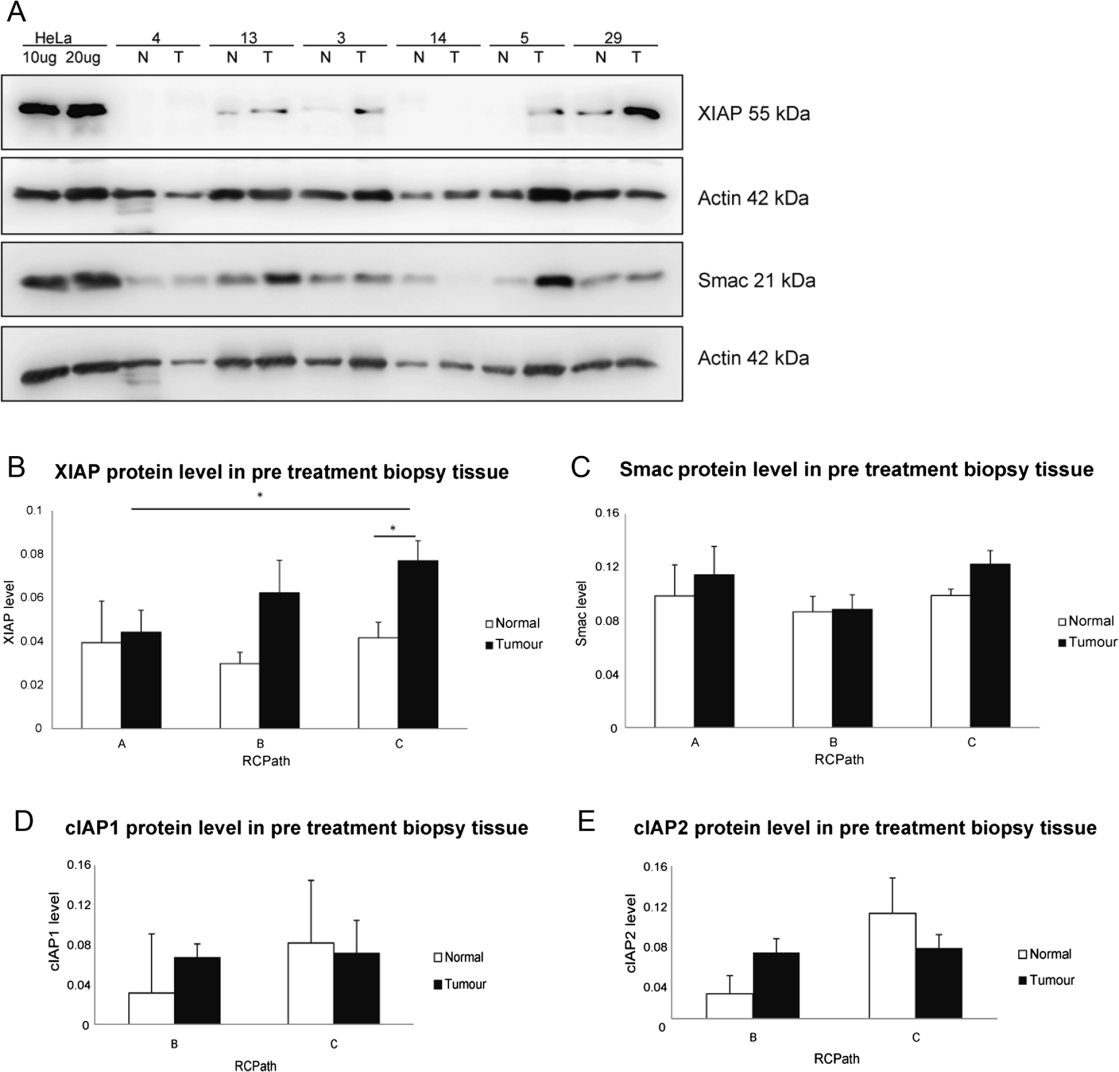
Elevated XIAP protein levels prior to neoadjuvant radio chemotherapy indicate resistance to therapy in rectal cancer patients. Biopsy tissue samples of 29 rectal cancer patients were obtained at colonoscopy or rigid sigmoidoscopy. **a** Representative Western blot images for 6 patients (3 RCPath A, 2 RCPath B and 1 RCPath C) are shown in Fig. 2a. The expression levels of XIAP and Smac in tumour (T) and matched normal tissue (N) were determined by quantitative Western blotting. Beta Actin was used as a loading control. HeLa cells were used as a standard. **b** Protein expression levels for all 29 patients were then correlated with tumour regression grade. Levels of XIAP protein increased in accordance with radio chemotherapy resistance (*p* = 0.025) in tumour tissue. XIAP protein levels are also significantly raised in the tumour tissue compared to the matched normal tissue in RCPath C patients (*p* = 0.007). **c** Smac protein was not differentially expressed in RCPath grades. Furthermore there was no significant difference in Smac expression in tumour and matched normal tissue. (**d**, **e**) Expression levels of cIAP-1 (**d**) and cIAP-2 (**e**) were also determined by quantitative Western blotting in 14 biopsy tissues and then correlated with tumour regression grade. Neither protein was differentially expressed in RCPath grades nor was there a significant difference in expression in tumour and matched normal tissue

Pre-treatment biopsy tumour and matched normal tissue samples were obtained at colonoscopy or rigid sigmoidoscopy (Fig. 1) from 29 rectal cancer patients. Using quantitative Western blotting, the expression levels of XIAP and Smac in tumour and matched normal biopsy tissue were determined in all 29 patients. Representative Western blot images for 6 patients (3 RCPath A, 2 RCPath B and 1 RCPath C) are shown in Fig. 2a. Beta Actin was used as a loading control. HeLa cells were used as a standard as previously described [28] (Fig. 2a). Clinical data was then examined and protein levels were correlated to RCPath grade (Table 1).

When we examined expression levels in all 29 patients we see that as tumour tissue became more radio chemotherapy resistant, from RCPath A to RCPath C, XIAP levels significantly increased (*p* = 0.025) (Fig. 2b). Conversely Smac levels did not increase with RCPath grade (Fig. 2c). This indicated a shift in the expression of these two proteins as tissue becomes more resistant to therapy. While this pattern was very evident in tumour tissue, matched normal tissue did not follow this trend. In normal tissue both XIAP and Smac levels remained unchanged throughout the varying grades of radio chemotherapy resistance. In RCPath C patients XIAP protein levels were significantly raised in the tumour tissue compared to the matched normal tissue (*p* = 0.007). These data demonstrated the importance of the XIAP/Smac balance and as XIAP levels increase and outweigh Smac levels, cells may be able to avoid apoptosis, via increased caspase inhibition, and thus become more resistant to therapy. Due to limited availability of biopsy tissue cIAP-1 and-2 were examined in only 14 biopsy tissues. Of these tissues 2 were RCPath A, 4 were RCPath B and 8 were RCPath C. Consequently RCPath A and B were pooled and compared to RCPath C. Both cIAP-1 and-2 protein levels were similar in all patients regardless of pathological response to neoadjuvant radio chemotherapy, suggesting that these proteins do not play a contributory role when the XIAP/Smac balance is disturbed (Fig. 2d, e). When we investigated whether XIAP level in biopsy tissue may have predictive power in selecting patients who respond to therapy, we found that XIAP levels could predict patients that responded to therapy with a sensitivity of 80 % and a specificity of 88 %, outperforming TNM staging which could not predict patient response to therapy, with a sensitivity 20 % and a specificity 100 %.

### XIAP expression increases in tumour tissue during radio chemotherapy

Following neoadjuvant radio chemotherapy patients underwent surgery to remove any remaining tumour. In RCPath A patients, where a complete pathological response was achieved, no surgical resection tissue was available. In 8 RCPath B and C patients, where neoadjuvant radio chemotherapy induced only partial tumour regression, or no tumour regression respectively, surgical resection tissue was available post treatment (Fig. 1).

**Fig. 3:**
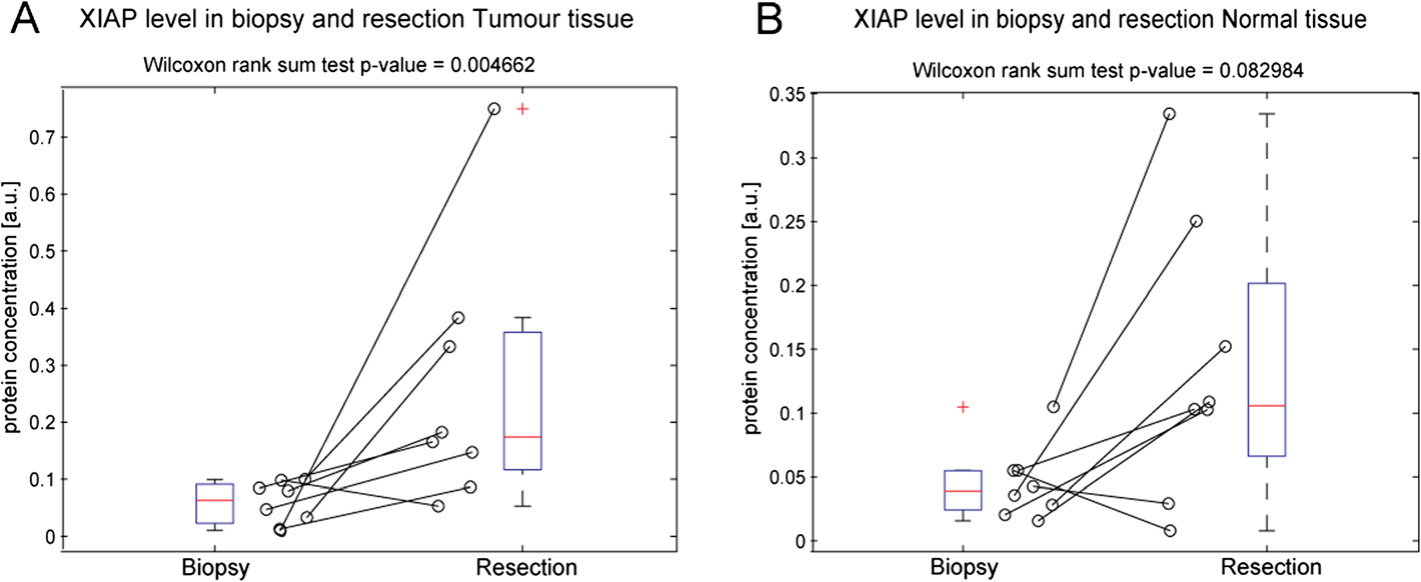
XIAP expression increases in tumour tissue during radio chemotherapy. For 8 patients where pre-treatment biopsy and matched post-treatment surgical resection tissue was available the expression levels of XIAP in tumour and matched normal tissue were once again determined by quantitative Western blotting. **a** In individual patients, all but one patient had increased XIAP levels in resection tumour tissue, compared to matched tumour biopsy tissue, and XIAP expression was significantly increased in post treatment resection tumour tissue, when compared to pre-treatment biopsy tumour tissue (*p* = 0.004662). **b** In matched normal tissue 6 out of 8 patients had increased XIAP levels in resection tumour tissue, compared to matched tumour biopsy tissue. This trend was not significant

**Fig. 4:**
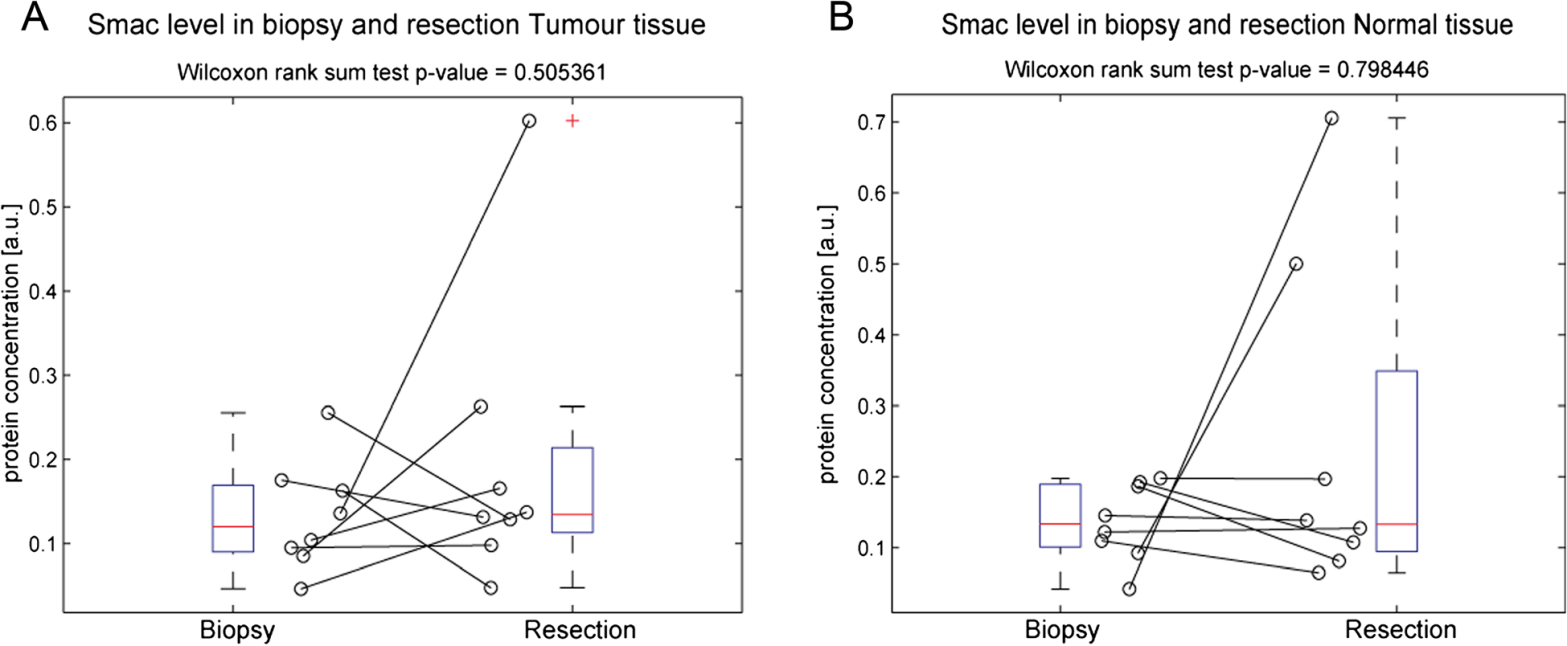
Smac expression does not change in normal and tumour tissue during radio chemotherapy. Smac expression levels in tumour and matched normal tissue were once again determined by quantitative Western blotting in pre-treatment biopsy and matched post-treatment surgical resection tissue, which was available for 8 patients. Smac expression was not significantly increased in post treatment resection (**a**) tumour and (**b**) normal tissue, when compared to pre-treatment biopsy tumour and normal tissue

**Fig. 5:**
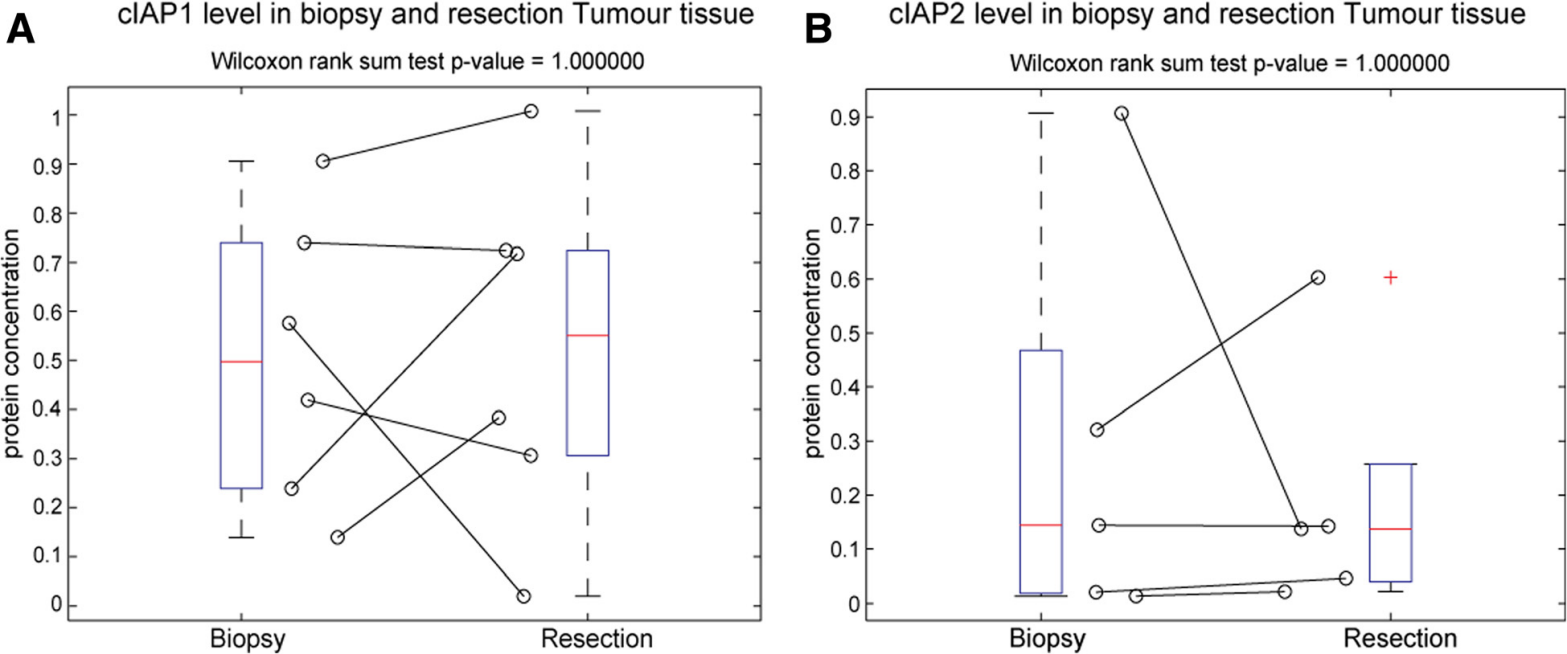
cIAP-1 and −2 expression does not change in normal and tumour tissue during radio chemotherapy cIAP-1 and −2 expression levels in tumour tissue were determined by quantitative Western blotting in pre-treatment biopsy and matched post-treatment surgical resection tissue. cIAP-1 was examined in 6 patients and cIAP-2 was examined in 5 patients. Expression of (**a**) cIAP-1 and (**b**) cIAP-2 was not significantly increased in post treatment resection tumour tissue, when compared to pre-treatment biopsy tumour and normal tissue

Using Western blotting we examined XIAP, Smac and cIAP-1 and-2 levels in these samples. XIAP levels were compared in pretreatment biopsy tissue and post treatment resection tissue. In tumour (Fig. 3a) XIAP expression increased significantly during the course of neoadjuvant radio chemotherapy (*p* = 0.004662). XIAP expression in matched normal tissue also increased but not significantly (*p* = 0.082984). Smac levels were also compared in pretreatment biopsy tissue and post treatment resection tissue (Fig. 4a, b). We saw no significant change in Smac expression in normal or tumour tissue pre and post treatment. This suggested that cancer cells that survive treatment responded by increasing their XIAP protein levels which may have resulted in radio chemotherapy resistance. Due to limited availability of tissue cIAP-1 and-2 was examined in tumour tissue only (Fig. 5a, b; cIAP-1 *n* = 6; cIAP-2 *n* = 5). Neither protein was affected by neoadjuvant radio chemotherapy, with no changes seen in expression levels in tumour tissue pre and post treatment.

## Discussion

In rectal cancer patients routinely undergo neoadjuvant radio chemotherapy. Radio chemotherapy, and also radiotherapy alone, prior to surgery has been shown to reduce rates of local recurrence and improve disease free survival, when compared to surgery alone [29, 30]. Patients who experience complete pathological response to neo-adjuvant radio chemotherapy experience low incidence of local recurrence and distant metastases [7, 8]. A response to neo-adjuvant radio chemotherapy of 95 % or greater is associated with a good long term outcome for the patient [31]. Many patients respond poorly to neoadjuvant radio chemotherapy but the reason for this is currently not well understood [9, 10, 32]. We examined XIAP, cIAP-1, cIAP-2 and Smac protein levels in a cohort of rectal cancer patients to examine whether more chemo resistant tissue displayed an altered protein expression. We found that XIAP levels in tumour tissue increased as chemo resistance grades progressed from RCPath A, through RCPath B, to RCPath C, suggesting XIAP may play a critical role in resistance to neoadjuvant radio chemotherapy. Furthermore our study highlights the potential of XIAP as a marker of response to neoadjuvant radio chemotherapy.

XIAP expression has previously been shown to be an important factor in cancer progression. In colorectal cancer cells high XIAP mRNA levels have been shown to be indicative of tumour differentiation, invasion and progression [33]. As well as contributing to disease progression XIAP was also reported to contribute to chemotherapy resistance [34]. In a cohort of 90 ovarian clear cell carcinoma patients high XIAP expression was found to correlate with lower chemotherapy response rates and also a worse progression free and overall survival for patients [19]. High levels of XIAP in lung cancer cells resulted in cisplatin mediated apoptosis resistance, and this apoptosis could be restored once XIAP was silenced [20]. Similar trends were reported in colorectal and breast cancer cell lines, where ceramide was found to effectively sensitise cells to apoptosis and also suppress tumour progression, via targeting of XIAP [18]. These studies highlight the importance of XIAP in cancer progression and demonstrate a crucial role for XIAP in triggering resistance to chemotherapeutic drugs.

Our work demonstrated that while XIAP expression increased as chemo resistance grades progressed from RCPath A, through RCPath B, to RCPath C, Smac levels remained constant. Smac is XIAP’s main antagonist, blocking XIAP’s caspase inhibitory function and allowing apoptosis to proceed. cIAP-1 and cIAP-2 are other inhibitor of apoptosis proteins that Smac binds to. As the XIAP/Smac balance shifts we saw no differential regulation of these proteins indicating that they play no contributory role when XIAP/Smac balance is disturbed. In many cancer cells as XIAP levels increase Smac levels do not mirror this pushing the cells into a more anti-apoptotic state. Findings similar to ours were previously reported in renal cell carcinomas (RCC). Yan et al. investigated XIAP and Smac expression in RCC, and found that Smac levels remained constant regardless of tumor progression, despite XIAP expression increasing significantly from early to advanced tumor stages. This disturbed XIAP/Smac balance may contribute to apoptotic resistance in RCC [25]. Another scenario where disruption of this protein balance was found to contribute to disease progression is Hodgkins disease. Hodgkins and Reed Sternberg cells and Hodgkin lymphoma derived B cells involved in this disease express high levels of XIAP. When the XIAP/Smac balance was restored in these cells they become re-sensitised to apoptosis [26].

As XIAP levels rise and the XIAP/Smac balance shifts Smac levels may not be sufficient to overcome XIAP’s anti-apoptotic action. Restoration of this balance may push cells back towards apoptosis and hence synthetic Smac peptides and small molecule Smac mimetics are being examined as novel cancer therapeutics. In combination with chemotherapy and radiotherapy these compounds have been reported to sensitise resistant cancer cells to apoptosis via modulation of both the apoptotic and NF-κB pathways [35–37]. The importance of the Smac/XIAP ratio in treatment responses to Smac mimetics was highlighted in childhood acute lymphoblastic leukaemia where XIAP is upregulated. Antagonism of XIAP, via a Smac mimetic, led to a significant increase in apoptosis in this setting [16]. Combined Smac mimetics and TRAIL treatment also reduced metastatic behaviour and cell migration [38]. Recent reports have highlighted that Smac mimetics can also trigger necroptosis as an alternative form of cell death to overcome apoptosis resistance in acute myeloid leukaemia cells [39]. Our data suggests that in the rectal cancer setting those patients who were unresponsive to traditional therapies and displayed high XIAP levels may benefit from Smac mimetic treatment to re-establish the XIAP/Smac balance, and in turn re-sensitise cells to therapy.

As well as increasing expression in radio chemotherapy resistant cells, we found that XIAP expression was also up regulated by neoadjuvant radio chemotherapy. Levels of XIAP in both normal and tumour tissue were significantly increased in post treatment surgical resection tissue, when compared to pretreatment biopsy tissue. Smac levels did not increase in response to therapy in either normal or tumour tissue, which once again contributes to a disruption of XIAP/Smac expression in cells. Recently it has been reported that chemotherapy induced enhanced XIAP expression, partially mediated through PI3K/Akt signalling, resulting in chemo resistance in breast cancer cells [40]. These data suggest that following treatment sensitive cells are killed off but a cohort of more resistant cells with high levels of XIAP remain. This has significant implications for further adjuvant treatment, as this more resistant population may continue to proliferate and be refractory to traditional therapies, highlighting another scenario where Smac mimetics would be an attractive alternative treatment regime.

## Conclusions

Our work highlights XIAP’s central role in resistance to neoadjuvant radio chemotherapy, leading to reduced therapy success. The use of Smac mimetics in this setting may restore the XIAP/Smac balance, in turn restoring apoptosis and leading to improved patient outcomes. Furthermore XIAP levels increase in response to neoadjuvant radio chemotherapy. Up regulation of this protein may lead to a more resistant phenotype in the adjuvant treatment setting, and patients defined by this XIAP over expression may benefit from alternative adjuvant treatment regimes, such as Smac mimetics.

## Abbreviations

XIAP: X-linked inhibitor of apoptosis protein
Smac: Second mitochondria-derived activator of caspases
TRAIL: Tumor necrosis factor-related apoptosis-inducing ligand
HRP: Horseradish peroxidase
T N M: Tumour, node, and metastasis
RCC: Renal cell carcinomas
5fu: 5-Fluorouracil
RCPath: Royal College of Pathologists
